# Rac1 Recruitment to the Archipelago Structure of the Focal Adhesion through the Fluid Membrane as Revealed by Single-Molecule Analysis

**DOI:** 10.1002/cm.21097

**Published:** 2013-03-05

**Authors:** Akihiro C E Shibata, Limin H Chen, Rie Nagai, Fumiyoshi Ishidate, Rahul Chadda, Yoshihiro Miwa, Keiji Naruse, Yuki M Shirai, Takahiro K Fujiwara, Akihiro Kusumi

**Affiliations:** 1 Institute for Integrated Cell-Material Sciences (WPI—iCeMS), Kyoto UniversityKyoto, Japan; 2 Institute for Frontier Medical Sciences, Kyoto UniversityKyoto, Japan; 3 Department of Pharmacology, Institute of Basic Medical Sciences, University of TsukubaIbaraki, Japan; 4 Cardiovascular Physiology, Okayama University Graduate School of Medicine, Dentistry, and Pharmaceutical SciencesOkayama, Japan

**Keywords:** single fluorescent-molecule imaging/tracking, PIX, diffusion, archipelago architecture, percolation

## Abstract

The focal adhesion (FA) is an integrin-based structure built in/on the plasma membrane (PM), linking the extracellular matrix to the actin stress-fibers, working as cell migration scaffolds. Previously, we proposed the archipelago architecture of the FA, in which FA largely consists of fluid membrane, dotted with small islands accumulating FA proteins: membrane molecules enter the inter-island channels in the FA zone rather freely, and the integrins in the FA-protein islands rapidly exchanges with those in the bulk membrane. Here, we examined how Rac1, a small G-protein regulating FA formation, and its activators αPIX and βPIX, are recruited to the FA zones. PIX molecules are recruited from the cytoplasm to the FA zones directly. In contrast, majorities of Rac1 molecules first arrive from the cytoplasm on the general inner PM surface, and then enter the FA zones via lateral diffusion on the PM, which is possible due to rapid Rac1 diffusion even within the FA zones, slowed only by a factor of two to four compared with that outside. The constitutively-active Rac1 mutant exhibited temporary and all-time immobilizations in the FA zone, suggesting that upon PIX-induced Rac1 activation at the FA-protein islands, Rac1 tends to be immobilized at the FA-protein islands. © 2013 Wiley Periodicals, Inc

## Introduction

**Fig. 1:**
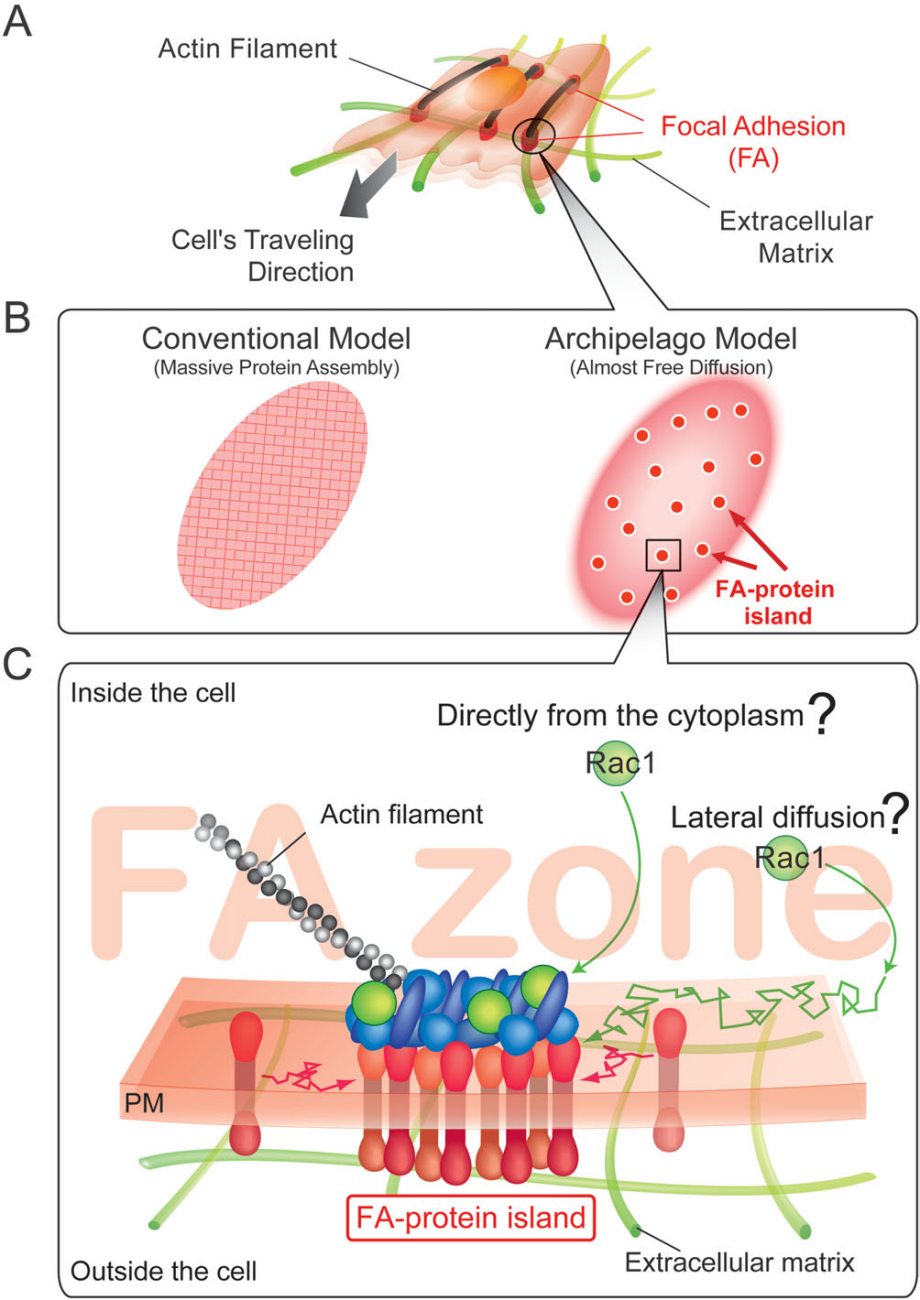
The working model of the archipelago structure of the FA and the recruitment processes of Rac1 and PIX molecules to FA-protein islands in the FA zone **(Panel A)** The FAs (red ellipsoids linked by the actin filament bundles in black) provide key scaffolds for the cells to migrate in/on the extracellular matrix (green filaments). **(Panel B)** Conventionally, the FA was considered to be a micron-scale massive assembly of various proteins (**Conventional Model**). However, our recent results [Shibata et al.,^2012^] indicated that the FA region is basically a fluid membrane region dotted with many much smaller islands made of the assembly of various FA proteins (FA-protein islands). Sufficient distances are maintained between the FA-protein islands in the FA zone, which allow the diffusion-based exchanges of membrane molecules between the FA zone and the bulk PM (**Archipelago Model**). **(Panel C)** The expanded structure of a part of the FA zone including an FA-protein island (top region, cytoplasm; bottom region, extracellular space), showing the pathways of Rac1 recruitment to the FA-protein island. Rac1 molecules might be recruited from the cytoplasm (where the majority of Rac1 molecules reside) to the FA-protein islands directly or might arrive on the cytoplasmic surface of the general PM area and then recruited to the FA-protein island via lateral diffusion in/on the inner PM surface (like integrins, red transmembrane molecules).

The focal adhesion (FA) is an integrin-based structure built in and on the plasma membrane (PM), mechanically linking the extracellular matrix with the termini of actin stress-fibers, providing key scaffolds for the cells to migrate in tissues (Fig. 1A) [Galbraith et al.,^2007^; Petrie et al.,^2012^]. Migrating cells form FAs in anterior regions and disassemble them in posterior areas, that is, the cell migrates on the extracellular matrix by repeatedly assembling and disassembling the FAs quickly in several to several 10 s of minutes [Betzig et al.,^2006^; Nayal et al.,^2006^; Shroff et al.,^2008^; Burnette et al.,^2011^]. Meanwhile, the FA had been considered as a micron-scale, massive assembly of various proteins (“conventional model” in Fig. 1B). How such large protein complexes in and on the PM can be formed or disintegrated in such short time periods has been a major mystery in cell biology. Furthermore, even in steadily existing FAs, the component molecules, such as integrins, were found to exchange with the molecules located in the bulk membrane, on a time scale of 30 s, which is unlikely if the FA is a single entity of huge protein assembly on the order of several microns [Goetz et al.,^2008^].

Recently, using single fluorescent-molecule imaging, we found that transferrin receptor and Thy1, non-FA membrane proteins, readily enter the FA zone, diffuse rapidly there, and exit into the bulk PM [Shibata et al.,^2012^]. Integrin β3 also readily enters the FA zone, and repeatedly undergoes the cycles of temporary immobilization and diffusion in the FA zone, whereas ∼1/3 of integrin β3 becomes immobilized there [Shibata et al.,^2012^; also see a follow-up study by Rossier et al.,^2012^]. Based on these observations, we proposed the archipelago architecture of the FA (“Archipelago Model” in Fig. 1B), or rather the FA zone, in which many small islands of integrins and other integrin-associated molecules (FA-protein islands) exist that are surrounded by much larger fluid-state (sea) area in the FA zone. The membrane molecules enter the inter-island channels rather freely by diffusion, and the integrins in the FA-protein islands can be rapidly exchanged with those in the bulk membrane. Therefore, such an archipelago architecture would allow rapid FA formation and disintegration.

Others also found evidence that the FA is not a single entity of massive protein assembly. Using photoactivated localization microscopy (PALM) with a 60-nm resolution, Shroff et al. [2008] found that the FA is subdivided in a manner resembling the city plan of Venice, where the land is subdivided by the narrow canals crisscrossing the city. A cryo-electron tomography investigation by Patla et al. [2010] revealed that actin stress-fibers are linked to membrane-bound particles of 25 nm in diameter existing at a density of 200/μm^2^ in small zones. However, since our previous results showed that transferrin receptor, Thy1, and integrin β3 readily enter the FA zone, diffuse rapidly there, and exit into the bulk PM (integrin β3 underwent repeated temporary immobilization and diffusion in the FA zone, and ∼1/3 of integrin β3 finally became immobilized there), we concluded that the areal fraction occupied by the islands in the FA zone must be less than 35% (a value called percolation threshold [Saxton,^1982^]); if the land area occupied more than this threshold value, such as the case of the Venetian city plan (where canals occupy very small fraction), the molecular diffusion in the FA zone would be reduced dramatically.

In the present investigation, we examined how signaling molecules that regulate FA formation, Rac1 and its activator αPIX and βPIX, are recruited to the FA domain [Chang et al.,^2007^] (Fig. 1B). Rac1, a small G-protein, regulates a signal transduction pathway linking membrane receptors to the assembly and disassembly of the FA and its associated actin cytoskeleton [Hall,^2005^; Xia et al., 2012]. αPIX and βPIX are guanine nucleotide (GDP-GTP) exchange factors (GEFs) that activate Rac1 via their SH3 binding to the proline-rich sequence of Rac1, inducing cell migration as well as tumor metastasis [Kuo et al.,^2011^; Hua et al.,^2011^]. Rac1 (mostly in the form bound by RhoGDI [Hancock and Hall,^1993^; Michaelson et al.,^2001^]) and PIXs are mostly located in various locations in the cytoplasm, but recruited to the PM for regulating various PM functions [del Pozo et al.,^2004^; Hoefen and Berk,^2006^; Chae et al.,^2008^]. Like K-Ras [Hancock et al.,^1990^; Michaelson et al.,^2001^; Parton and Hancock,^2004^; Hancock and Parton,^2005^], Rac1 is anchored to the PM by way of the polybasic peptide sequence and a hydrophobic chain [Silvius et al.,^2006^], with possible involvement of cholesterol [del Pozo et al.,^2004^] and phosphatidic acid [Chae et al.,^2008^], and is expected to undergo translational diffusion on the cytoplasmic surface of the PM [Niv et al.,^2002^; Kenworthy et al.,^2004^].

Our results indicate that whereas αPIX and βPIX are recruited from the cytoplasm to the FA zone directly, majorities of Rac1 molecules, including the wild-type (WT), constitutively-active (CA) G12V, and dominant-negative (DN) T17N Rac1 molecules, are recruited to the FA zone (from the cytoplasm) by way of the general PM area via lateral diffusion on the PM. Namely, many Rac1 molecules first arrive from the cytoplasm on the cytoplasmic surface of the general PM area, and then enter the FA zone by way of lateral diffusion on the inner PM surface. Such Rac1 recruitment to the FA zone is possible because Rac1 molecules can undergo translational diffusion within the FA region, that is, the diffusion coefficients of Rac1 within the FA zone is slower than that outside the FA zone only by a factor of two to four. Meanwhile, the CA mutant G12V often undergoes frequent, intermittent temporary immobilization as well as total immobilization in the FA zone, suggesting that when Rac1 is activated in the FA domain by PIX, which is concentrated and immobilized in the FA zone, probably at the island of FA-associated molecules, Rac1 becomes more prone to immobilization at the FA-protein islands.

## Results

### Simultaneous Observations of the FA Zone and Single Molecules of Rac1 or PIX

**Fig. 2:**
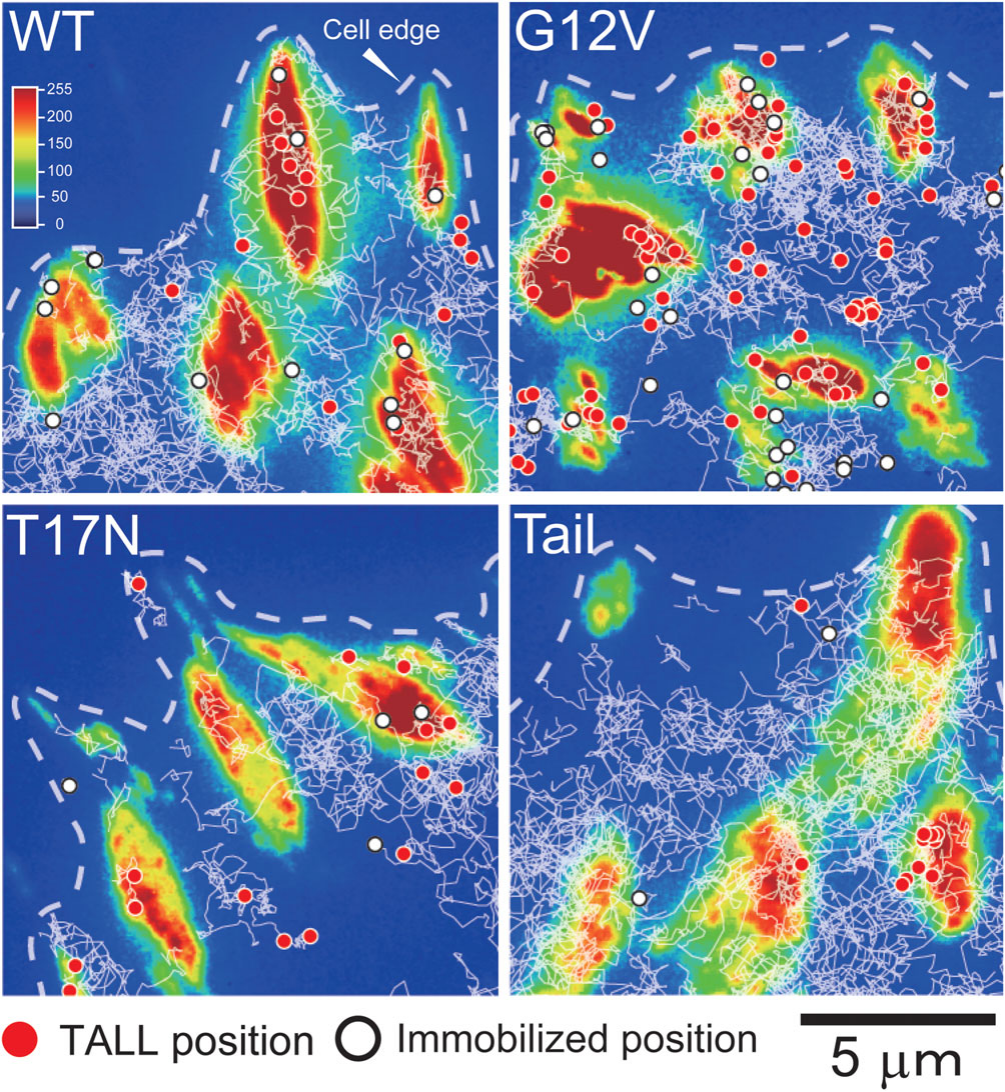
Rac1 molecules can diffuse into the FA zone The images of mGFP-paxillin (its signal intensity, representing its number density, is color coded, see the scale in the box on the top) and typical trajectories of WT, G12V, T17N, and Tail, obtained by single fluorescent-molecule imaging at a frame rate of 60 Hz, are superimposed. Whereas many of these four types of Rac1-related molecules enter the FA zone and undergo diffusion there, many G12V molecules also frequently exhibited temporary (red circles) and long-term (white circles) immobilizations.

**Fig. 3:**
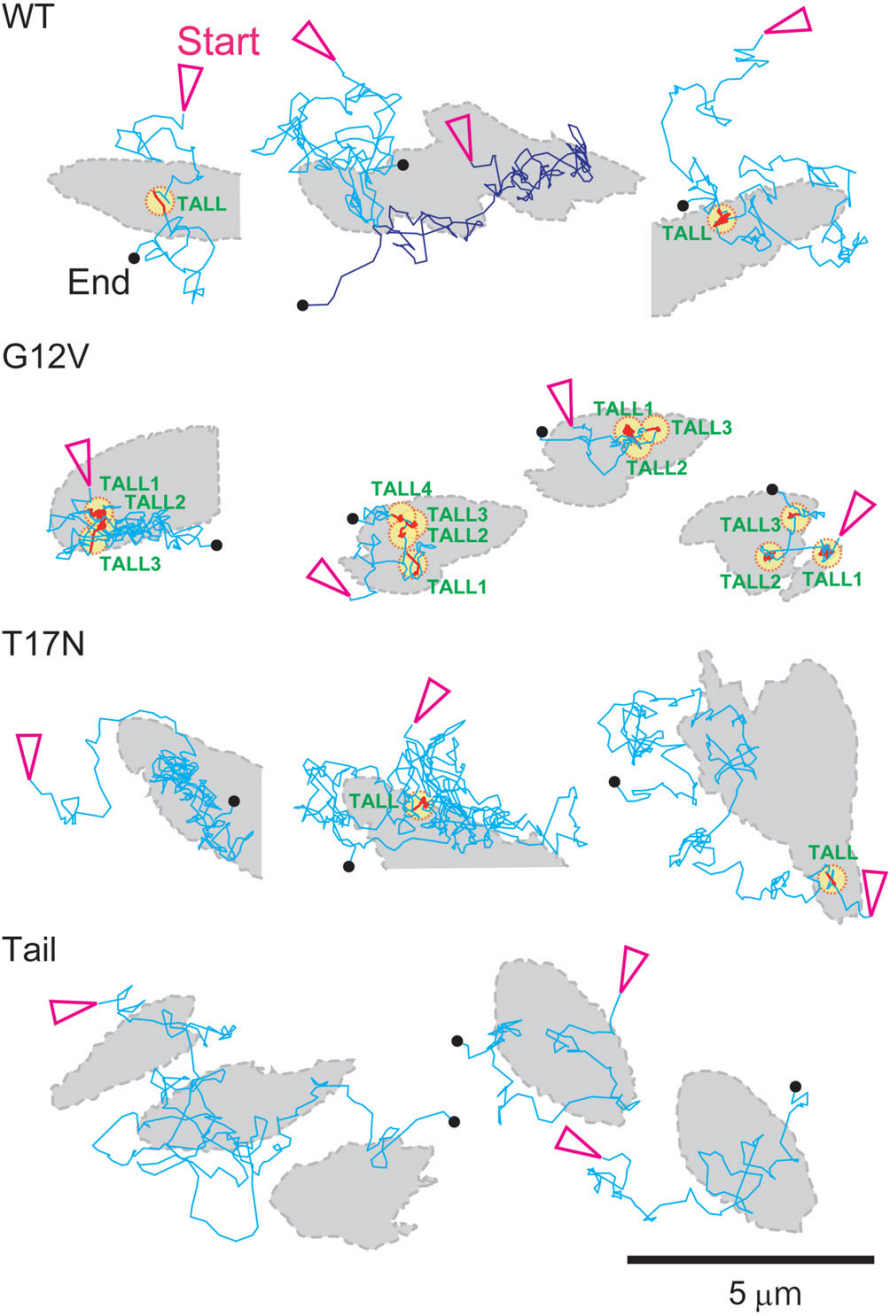
Rac1 molecules readily enter and exit from the FA zone Binarized images of mGFP-paxillin (shown by grey shading with dashed outlines, see Materials and Methods) and typical single-molecule trajectories of WT, G12V, T17N, and Tail, obtained at a frame rate of 60 Hz, are superimposed. The trajectories of molecules immobilized throughout the observation period (until they became photobleached) were not included here. G12V exhibited TALL behaviors much more frequently than WT or T17N (TALL trajectories and locations are marked in red and yellow, respectively. When a molecule exhibited TALL more than once, the order of TALL events is indicated by the number after the word TALL).

HeLa cells transfected with appropriate cDNAs were cultured on coverslips of glass-base dishes on which fibronectin had been printed (1.5 × 4.5 μm ellipse, with 3-μm spacings for both *x* and *y* directions). Since, by using the micro-printed fibronectin, FA zones tended to exhibit similar shapes and sizes, the analysis of the experimental results was facilitated (the FA zones near the cell edges tended to be greater than the printed ellipse size, but those in the middle of the cells exhibited the same shape as the micro-printed fibronectin). The micro-printed fibronectin did not affect the mobility of Thy1 in the FA domain [Shibata et al.,^2012^]. FA domains were fluorescently marked and imaged, by conjugating the mGFP to the N-terminus of the FA's molecular scaffold protein paxillin (mGFP-paxillin), which is frequently used for imaging FAs [Brown and Turner,^2004^; Shibata et al.,^2012^]. mGFP-paxillin was observed at multimolecular level using the TIRF microscopy (Fig. 2). The mGFP-paxillin images were binarized, and then the boundaries of the FA zones were determined, as previously described by Shibata et al. [2012] (Fig. 3; Materials and Methods). In the following part of this report, the FA zones are defined as those determined by such binarization.

Rac1 or αPIX conjugated to the Halo-Tag protein at their N-termini (Halo-Rac1 or Halo-αPIX, respectively) or βPIX at its C-terminus conjugated to the N-terminus of the Halo-Tag protein (βPIX-Halo) was coexpressed with mGFP-paxillin and was fluorescently labeled with the Halo ligand conjugated with tetramethylrhodamine (TMR), and observed at the single molecule level. For the sake of simplicity, in the following part of this report, Halo-Rac1, Halo-αPIX, or βPIX-Halo will be simply called Rac1, αPIX, and βPIX, respectively, wherever their meanings are clear.

Using a home-built TIRF microscope for simultaneous two-color single-molecule observations [Koyama-Honda et al.,^2005^], we alternatingly imaged (every few seconds; see Materials and Methods) single molecules of one of the membrane-associated molecules and the bulk mGFP-paxillin, at an observation frame rate of 60 Hz (a time resolution of 16.7 ms, respectively). In the present work, all of the observations were made in the basal PMs of HeLa cells at 37°C.

### Where do Rac1, αPIX, and βPIX Molecules First Land on the Inner Surface of the PM, When they Arrive from the Cytoplasm?

Large majorities of Rac1, αPIX, and βPIX molecules are located in the cytoplasm, and they are recruited to the PM for their functions at the PM. The arrival of these molecules from the cytoplasm at the PM was investigated at the single molecule level. They undergo three-dimensional thermal diffusion in the cytoplasm, and by chance they arrive at the inner surface of the PM. When they collide with the inner surface of the PM and instantaneously leave there, within less than two frames of the detecting camera, they will not form images or even when they form images in a single camera frame, those images are difficult to be distinguished from the shot noise of the camera. Therefore, we can detect single Rac1, αPIX, and βPIX molecules when they stayed on the cytoplasmic surface of the PM at least for two frames, that is, 33 ms. In addition, in the present research, we found that the the distribution of the residency times of Rac1 on the inner PM surface could *operationally* be fitted with the sum of two exponential decay functions, with lifetimes of 0.17 and 1.53 s (distribution not shown). Since we are interested in Rac1 molecules that stay longer on the cytoplasmic surface, for the analysis in this study, we only employed molecules that stayed on the PM longer than 11 consecutive image frames (183 ms, which is given by the number of frames [11], which is the smallest number of frames longer than 0.17 s) as those attached to the PM (Supporting Information Movies S1, S2, and S3).

**Fig. 4:**
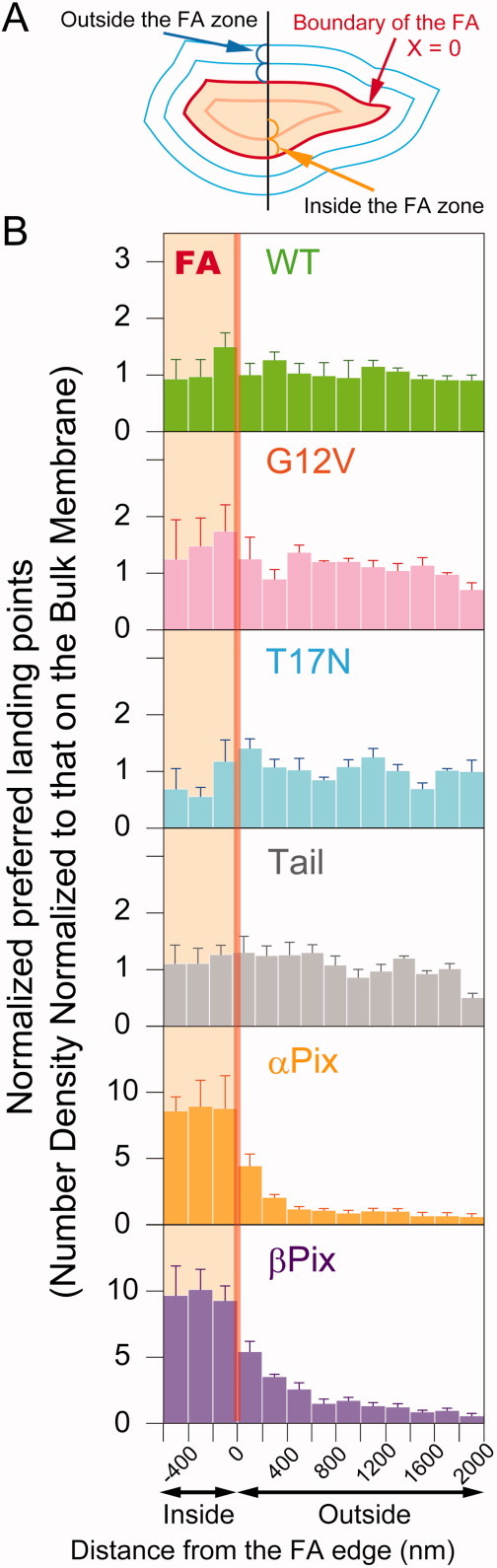
The distributions of the landing points (the places where Rac1 [WT, G12V, T17N, and Tail] and α/βPIX first arrived) on the cytoplasmic surface of the PM, from the cytoplasm, with respect to the FA zone **(Panel A)** A schematic drawing showing an FA zone, its boundary (*x* = 0), and 200-nm-wide strips parallel to the FA boundary (see Materials and Methods). The PM was divided into zones of every 200 nm in width surrounding and inside the FA zone. Zero on the *x*-axis is set at the FA boundary. **(Panel B)** Normalized preferred landing points on the inner PM surface for Rac1 and PIX molecules, with respect to the FA zone boundary. The distributions of the locations where Rac1 and PIX molecules first arrived from the cytoplasm on the inner PM surface are shown as the number densities of the arrival locations, normalized to their average values well outside the FA zone (1000–2000 nm; shown as the mean ± SE for three totally independent experiments). The orange regions in the histograms indicate the areas inside the FA.

The distributions of the locations where Rac1 (WT, CA, and DN), αPIX, and βPIX first arrived on the cytoplasmic surface of the PM, from the cytoplasm, with respect to the FA zone are shown in Fig. 4. Note that the *y*-axis represents the number density of landing locations normalized to that on the bulk PM area.

Full-length Rac1 molecules, including WT as well as CA and DN mutants, that stayed longer than 183 ms on the PM arrive on the PM nonspecifically (Fig. 4). In contrast, αPIX and βPIX molecules that stayed longer than 183 ms on the PM preferrentially arrive in the FA zone: PIX molecules should arrive at the PM virtually anywhere equally, but great majority of them leave the surface instantaneously, but some of the molecules stayed longer than 183 ms, and the probability in which αPIX and βPIX molecules stayed longer than 183 ms was much higher in the FA zone compared with that in the bulk PM region by a factor of approximately 9 on average (Fig. 4). This result suggests that Rac1 and PIX molecules are recruited to the FA zone by way of different pathways.

### Rac1 Molecules Enter FA Zones by Translational Diffusion on the PM Cytoplasmic Surface and Undergo Complex Behaviors in FA Zones

Based on the archipelago architecture model of the FA zone, we examined the possibility, in which Rac1 molecules that landed on the inner surface of the general PM area is recruited to the FA zone by translational diffusion on the PM surface. Single molecules of WT-Rac1 underwent diffusion in the PM, and upon the encounter with an FA zone, they often entered the FA zone, exhibited diffusion within the FA zone, and then exited from the FA region (Figs. 2 and 3 and Supporting Information Movie S1). In addition, some of the WT-Rac1 molecules exhibited *T*emporary *A*rrest of *L*atera*L* diffusion (TALL; we use this term because we previously introduced the term *S*timulation-induced TALL or STALL [Suzuki et al.,2007a, b]). The G12V mutant exhibited TALL events more frequently, whereas the WT exhibited much less TALL behavior (Figs. 2 and 3; quantitative data described in the following paragraphs and in Table I Top and Supporting Information Movie S2).

**Table I:** The Time Fractions of Mobile, TALL, and Immobile Periods for WT, G12V, T17N, and Tail Inside and Outside the FA Zone

	Time fraction (%)					

Molecule	Mobile	TALL	Immobile	τ_TALL_ (s)	*n*_molecule_	Total (s)
Inside FA Zones						
WT	65	10	25	0.21	128	43.0
G12V	29	36	35	0.48	168	119.0
T17N	72	21	7	0.41	195	81.4
Tail	78	7	15	ND^a^	319	108.7
αPix	1	4	95	ND^b^	1260	2141.7
βPix	1	4	95	ND^b^	1332	2420.0
Outside FA Zones						
WT	72	11	17	0.22	914	650.0
G12V	59	27	14	0.20	1144	1163.8
T17N	88	9	3	0.19	1107	671.6
Tail	89	6	5	ND^a^	1499	938.6
αPix	6	12	82	ND^b^	1247	1911.2
βPix	6	15	79	ND^b^	1332	2376.2

a Not determined due to the lack of the second, longer component in the double exponential fitting of the TALL duration histogram (Supporting Information, Fig. S1).

b Not determined due to the difficulties in fitting the distribution using the sum of two exponential functions.

The parts of single-molecule trajectories of Rac1 that were either inside or outside the FA zone (only those longer than 11 consecutive frames or 183 ms, providing ≥10-steps- or ≥167-ms-long trajectories, were employed for further analysis in this study, unless otherwise stated) were classified into three modes of motion: (1) the all-time mobile mode, (2) the mobile + TALL mode in which a mobile trajectory includes at least one TALL period, and (3) the all-time immobile mode. This categorization was performed by using the algorithm developed by Sahl et al. [2010] and used extensively by Shibata et al. [2012]. Next, the trajectories that were classified into the all-time mobile mode and the mobile parts of the trajectories that were classfied into the mobile + TALL mode were pooled, to be analyzed as trajectories of mobile molecules.

Even within the FA zone, the time fractions of the mobile periods for WT and T17N (a DN mutant) as well as the lipid-anchoring tail domain of Rac1 (called Tail, which contains the basic peptide sequence VKKRKRKCLLL with a geranylgeranyl anchor at the C-terminus for the PM anchoring [Michaelson et al.,^2008^] and the Halo protein [TMR-conjugated] at the N-terminus for visualization) remained high (70–80%, Table I) and comparable to the mobile time fractions outside the FA zone (70–90%, Table I). Note that we use time fractions rather than molecular fractions in this report. Due to the release of Rac1 and its mutants from the PM into the cytoplasm (for example, immobile molecules tend to stay on the membrane surface longer), the behavior of Rac1 molecules on the cytoplasmic surface of the PM can be better quantitated using time fractions rather than molecular fractions.

Meanwhile, the G12V (a CA mutant) trajectories exhibited high proportions of TALL (36%) and all-time immobilization (during the observation period, 35%; namely ≍1/3 of G12V molecules we observed were already immobilized in the FA zone at the onset of our observations and remained immobile throughout the observation periods) inside the FA zone (Table I). The all-time immobilization and TALL of G12V inside the FA zone might represent G12V's binding to the FA-protein islands (which are, in turn, conjugated to actin stress-fibers, Figs. 1A and 1C). The larger time fractions of TALL + all-time immobile durations of G12V compared with those of WT and T17N indicates that the activated Rac1 binds to the FA-protein isands much more effectively. When WT molecules enter the FA zone and if they are activated by PIX molecules (GEFs), they might become more susceptible to immobilization by the islands of FA proteins. However, pay attention to the result in which the lifetime of each TALL event was short (exponential decay time = 0.48 s for G12V; see Supporting Information, Fig. S1). Namely, mobile G12V molecules (64% of all G12V molecules) repeatedly undergo TALL and diffusion within the FA zone (∼1:1 temporal fraction).

The TALL time fraction of T17N within the FA zone is higher than that of WT by a factor of about two. This might be due to T17N's (transient) binding to its GEF, perhaps including PIXs [ten Klooster et al.,^2006^; Chang et al.,^2007^], because its related molecule T17N-*Ras* exhibited a dissociation constant with its GEF ∼2000 times lower than WT-*Ras* [Feig,^1999^]. Furthermore, T17N does not bind to RhoGDI [Michaelson et al.,^2001^], nor its downstream molecule PAK [Nomanbhoy and Cerione,^1996^].

WT and G12V exhibited immobilization and TALL even outside the FA zone (30–40% time fraction). This could be due to their binding to structures other than FA (perhaps such as the actin-based membrane skeleton) and/or small FA zones that might not be readily visible due to their faint mGFP-paxillin signals (Table I Bottom).

### Even Inside the FA Zone, Mobile Rac1 Molecules Diffuse Rapidly at Rates Slowed by a Factor of Two to Four

**Fig. 5:**
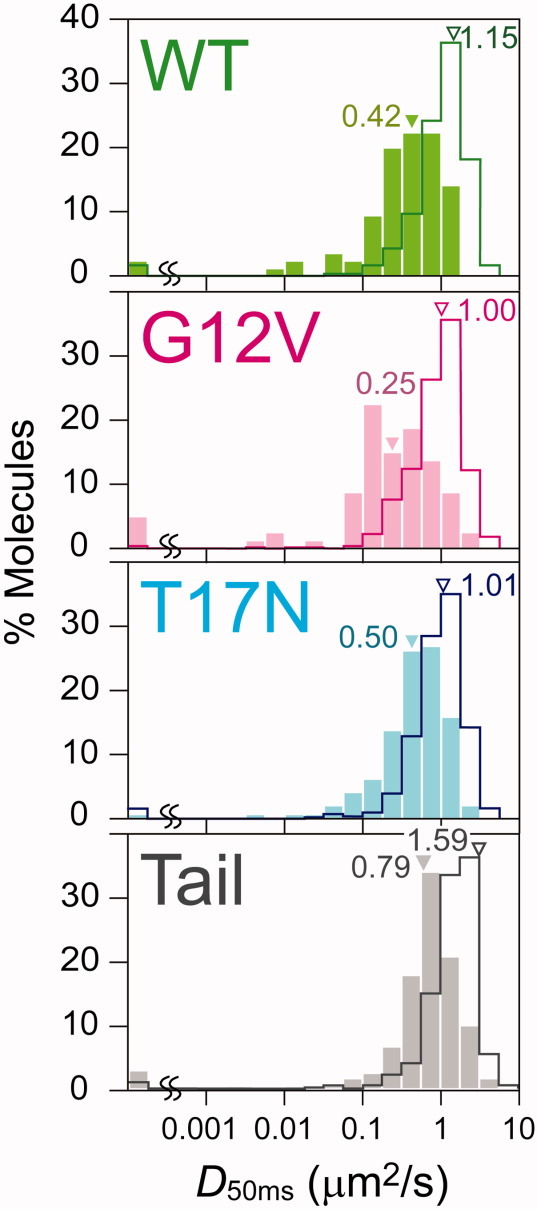
Even inside the FA zone, the mobile molecules of Rac1 and its mutants diffused rapidly, at rates only 2–4-fold slower than those outside the FA zone The distributions of the diffusion coefficients in the time scale of 33–67 ms (*D*_50ms_) of WT, G12V, T17N, and Tail inside (closed bars) and outside (open bars) the FA zone, determined for trajectories classified into the all-time mobile mode. The values in the boxes indicate median values. See Table II for the summary of the median and mean ± SE values.

The distributions of the diffusion coefficients (in the time scale between 33 and 67 ms, *D*_50ms_) for single molecules of Rac 1 (WT, G12V, T17N, and Tail) during the mobile period are shown in Fig. 5 (the summary of diffusion coefficients *D*_50ms_s provided in Table II). Outside the FA zone, the full-length Rac1 molecules diffused at similar rates with a diffusion coefficient of ∼1.1 μm^2^ ·s^−1^ (an average of *D*_50ms_s for WT, G12V, and T17N), whereas the Tail molecules diffused faster. Perhaps due to the smaller size of the protein domain of Tail compared with that of the full-length Rac1, the effect of actin-based membrane-skeleton fence in suppressing the diffusion was smaller on Tail than full-length Rac1 molecules [Kusumi et al.,^2011^,^2012^].

**Table II:** The Diffusion Coefficients Estimated on the Time Scales of 33–67 ms (*D*_50ms_ μm^2^/s) for WT, G12V, T17N, and Tail Inside and Outside the FA

	Wild-type	G12V	T17N	Tail
Inside FA	0.42	0.25	0.50	0.79
	0.51 ± 0.043	0.42 ± 0.056	0.62 ± 0.039	0.95 ± 0.050
Outside FA	1.15	1.00	1.01	1.59
	1.36 ± 0.14	1.11 ± 0.034	1.13 ± 0.025	1.69 ± 0.033

Values for the trajectories classified into the all-time mobile mode. Median (top) and Mean ± SE (bottom) values are listed.

Inside the FA zone, the diffusion coefficients of the full-length Rac1 and Tail molecules were reduced, but the extent of reduction was limited to a factor of 2–4 from those outside the FA zone (Fig. 5 and Table II, estimated for trajectories classified into the all-time mobile mode). More precisely, the diffusion coefficient of WT was reduced within the FA zone by a factor of three, that of G12V by a factor of four, and those of T17N and Tail by a factor of two. The small reduction of the diffusion coefficient of Tail, which is a molecule that will not associate with any specific FA proteins, is consistent with the archipelago model of FA in that membrane molecules can enter the FA zone rather freely and diffused even within the FA zone, but not with the Venetian city plan model. The Venetian city plan and archipelago models are distinguished based on the ability of membrane molecules to undergo macroscopic diffusion in the FA zone. In the archipelago model, *membrane molecules undergo macroscopic diffusion at a rate comparable to that outside the FA zone*. Meanwhile, in the Venetian city plan model, the macroscopic diffusion of membrane molecules is virtually blocked or considerably slowed (by an order of magnitude or more in the macroscopic diffusion coefficient), despite the presence of the fluid membrane (canals), due to its small areal fraction [Saxton,^1982^; Holcman et al.,^2011^]. More reductions in diffusion coefficients of WT and G12V within the FA zone relative to those of T17N and Tail suggest that WT and G12V often associate with FA proteins in the islands transiently (G12V would do more, considering greater reduction compared with the reduction seen with WT). Here, we are analyzing the diffusion coefficients of all-time mobile molecules, but shorter immobilization events due to transient association with the FA-protein islands could not be detected as TALL events and their trajectories were classified into the all-time immobile mode (with smaller diffusion coefficients).

### Rac1 Molecules Exit from the FA Zone in 0.24–0.81 s or Stay in the FA Zone

**Fig. 6:**
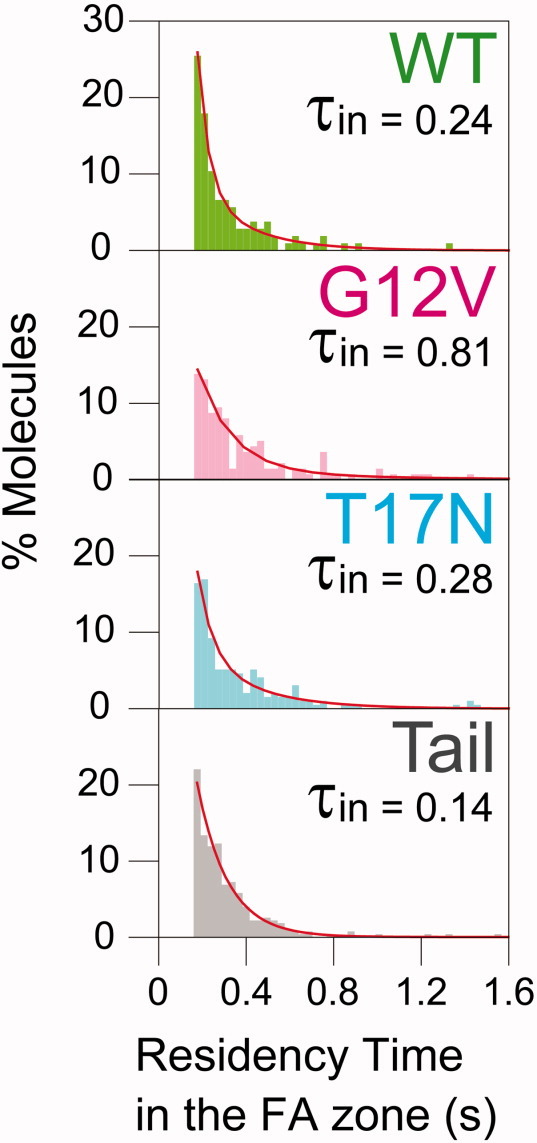
The residency times of Rac1 and its mutants within the FA zone are 0.14–0.81 s after correction for photobleaching The distribution of residency times determined for all molecules that entered and exited from the FA zone during the observation time. Each distribution was operationally fitted with the sum of two exponential functions: the long component was considered to represent the residency time, whereas the short component (<70 ms) was probably due to noise of single-molecule localization (when molecules approach the FA boundaries, due to noise, they appear to go back and forth across the FA boundaries). The obtained residency time was then corrected for photobleaching lifetime of TMR bound to the Halo protein (14.2 s), which is the value indicated in each box.

Except for the all-time immobile trajectories, some of the WT, G12V, and T17N molecules outside the FA zone entered FA zones and exited in 0.24–0.81 s (after correction for photobleaching lifetime, the exponential residency time as described in Fig. 6, summarized in Table III). Tail molecules exited even more quickly (since each view field was recorded for 16.7 s, the duration of observation would hardly affect the estimate of the residency times).

**Table III:** The Exponential Residency Times within the FA Zone (τ_in_) for the Full-Length Rac1 and Tail Except for the Molecules Immobilized within the FA Zone during the Entire Observation Period

Molecules	τ_in_ (s)	*n*_mol_	Total observation period (s)
WT	0.24	106	43
G12V	0.81	137	119
T17N	0.28	183	81
Tail	0.14	277	85

The analysis was performed for the molecules that remained within the FA zone longer than 11 frames (10 steps or 165 ms in trajectories). These values were corrected for the photobleaching lifetime of the TMR (14.2 s). For the derivation of the residency times, see Supporting Information, Fig. S1.

However, some molecules were immobile in the FA zone from the beginning of the recording and some became immobilized in the FA zone during the observation period (28 and 46% of the time fractions for WT and G12V molecules, respectively, counting both all-time immobile molecules and the molecules that exhibited TALL in the FA zone at the end of their trajectories). Approximately only 15% of T17N and Tail molecules were or became immobilized in the FA zone, which is much less than WT or G12V. These results suggest that when WT molecules became activated in the FA zone, they tend to become bound to and immobilized on the FA-protein island for longer periods.

Furthermore, the G12V molecules that entered and exited from the FA zone stayed in the FA zone much longer than WT and T17N molecules (0.81 s vs. ≍0.26 s). The longer residency of G12V in the FA zone could largely be explained by its frequent TALL events within the FA zone (Fig. 3 and Table I) and the reduced diffusion coefficient within the FA zone (Fig. 5), suggesting that activated Rac1 dynamically binds to the FA-protein islands. Meanwhile, presently, we do not understand why the residency time of T17N in the FA zone is about the same as that of WT. Perhaps, WT molecules are more immobilized rather than exhibiting TALL and temporary diffusion (25 vs. 7% for WT and T17N, as shown in Table I).

Considering the size of the FA zone (generally greater than 500 nm, see Figs. 2 and 3 for typical FA images), the short Rac1 residency times within the FA zone, despite Rac1′s frequent visits deep inside the FA zone (Fig. 3), show that the FA zone is not isolated from the bulk PM, consistent with the FRAP data [Goetz et al.,^2008^] and single-molecule tracking data of integrin molecules [Rossier et al.,^2012^; Shibata et al.,^2012^]. Taken together, these results suggest that the FA-protein islands exist rather sparsely in the FA zone, perhaps representing less than 10% of the FA zone area [Saxton,^1982^]. Otherwise, the central part of the FA zone would have been much less accessible by Rac1 and other membrane molecules, and their diffusion coefficients within the FA zone would have been decreased much more severely.

PIX residency times in the FA zone directly measured in these experiments were comparable to the photobleaching lifetime (≍ 14.2 s). Therefore, their true residency times after photobleaching lifetime could not be accurately determined, but longer than 14.2 s.

### Rac1 Recruitment to the FA Zone Occurs via Direct Binding to the FA Zone as Well as via Lateral Diffusion from the General Membrane Area

When Rac1, αPIX, and βPIX molecules located in the cytoplasm collide with the inner surface of the PM as a result of their three-dimensional diffusion in the cytoplasm, some of the molecules stay on the PM surface for longer than 33 ms and become visible by the present single-molecule observation method. When the molecules that stayed on the PM for longer than 183 ms were analyzed, Rac1 molecules initially stick to the inner PM surface nonspecifically (Fig. 2). In contrast, the αPIX and βPIX molecules preferrentially bind to the FA zone. This result suggests that Rac1 and PIX molecules are recruited to the FA zone by way of different pathways.

We further examined how molecules entered the FA zone for the first time (this analysis was limited to molecules that entered the FA zone at least once), that is, whether they entered via translational diffusion on the inner surface of the PM after binding to the general PM surface outside the FA zone or by directly binding to the FA domain from the cytoplasm (Table IV). Approximately 20% of Tail molecules arrived in the FA zone directly from the cytoplasm (i.e., 80% of Tail first bound to the general membrane area and then entered the FA zone via lateral diffusion on the inner PM surface), which would be a value representing the arrival of molecules that have no specific interaction with the molecules assembled in the FA zone. Meanwhile, approximately 30% of WT molecules arrived in the FA zone directly from the cytoplasm (i.e., 70% of WT first bound to the general membrane area and then entered the FA zone via lateral diffusion on the inner PM surface), suggesting that when Rac1 binds to the PM, the FA zone is only slightly preferred.

**Table IV:** Percentages of Molecules that Entered FA Zones via Lateral Diffusion after Binding to the PM Outside FA Zones Or Directly Arrived in the FA Zone from the Cytoplasm

Molecules	Via lateral diffusion on the PM (%)	Direct recruitment from the cytoplasm (%)
WT	70.2 ± 4.9	29.8 ± 4.9
G12V	68.1 ± 4.9	31.9 ± 4.9
T17N	75.2 ± 3.7	24.8 ± 3.7
Tail	78.2 ± 0.3	21.8 ± 0.3
αPix^a^	3.8 ± 1.5	96.2 ± 1.5
βPix^a^	5.0 ± 0.6	95.0 ± 0.6

a Values for αPix and βPix include those immobilized within the area between 0 and 200 nm from the edge of the FA zone (see the first bin in the histograms shown in Fig. 2) due to the following reason. Mostly due to noise (limited localization accuracies of single molecules), many molecules that directly arrived in this area and immobilized there apparently move back and forth between this area and the area between −200 nm and 0 nm from the FA edge. This distorts the count of molecules that entered the FA zone directly from the cytoplasm and via lateral diffusion on the PM.

In contrast, most of αPIX and βPIX molecules (96%, average of the two molecules, Table IV) entered the FA zone directly from the cytoplasm. Since 95% of αPIX and βPIX molecules in the FA zone are immobile (Table I), it is concluded that they bind to the FA-protein islands directly from the cytoplasm, become immobilized, and work there.

For the function of Rac1 in the FA zone (e.g., activating PAK), if Rac1 is bound to the islands of FA-associated molecuels, it might work more efficiently due to molecular concentration in the FA-protein islands. Therefore, we further perfomed the recruitment analysis by examining the recruitment pathways of the molecules that exhibited at least one TALL period (we examined molecules exhibiting any TALL or TALL longer than 0.48 s) inside the FA zone. Approximately half of such molecules (WT and G12V) entered the FA zone via lateral diffusion, whereas the other half entered the FA zone by direct binding from the cytoplasm (Table V). Taken together, these results indicate that both pathways would be important for Rac1 recruitment to the FA zone.

**Table V:** Percentages of Molecules that Entered FA Zones via Lateral Diffusion after Binding to the PM Outside FA Zones or Directly Arrived in the FA Zone from the Cytoplasm. Unlike the Values in Table IV, these Percentages were Separately Evaluated for Molecules that Exhibited at Least One TALL Period + the All-time Immobile Mode and for those Exhibited the All-time Mobile Mode

	Molecules that exhibited TALL and the all-time immobile mode		Molecules that exhibited the all-time mobile mode	
	
Molecules	Via PM	Direct	Via PM	Direct
All molecules that entered the FA zone (%molecules)				
WT	5.0 ± 0.2	6.4 ± 0.8	65.2 ± 4.8	23.4 ± 4.1
G12V	16.3 ± 1.1	13.0 ± 3.2	51.8 ± 5.1	18.9 ± 1.8
Tail	3.3 ± 0.9	2.0 ± 0.2	74.9 ± 1.1	19.8 ± 0.3
Molecules that exhibited TALL or immobile periods longer than 0.48 s in the FA zone (%molecules)				
WT	1.6 ± 0.7	2.1 ± 1.2	68.7 ± 4.9	27.7 ± 4.8
G12V	8.5 ± 1.3	5.5 ± 1.4	59.6 ± 5.7	26.4 ± 3.6
Tail	0.7 ± 0.1	0.8 ± 0.1	77.5 ± 0.3	21.1 ± 0.3

The period of 0.48 s was selected because it was the exponential lifetime of TALL events for G12V, which is constitutively active.

### Activated Rac1 might be Accumulated in the FA Zone

**Fig. 7:**
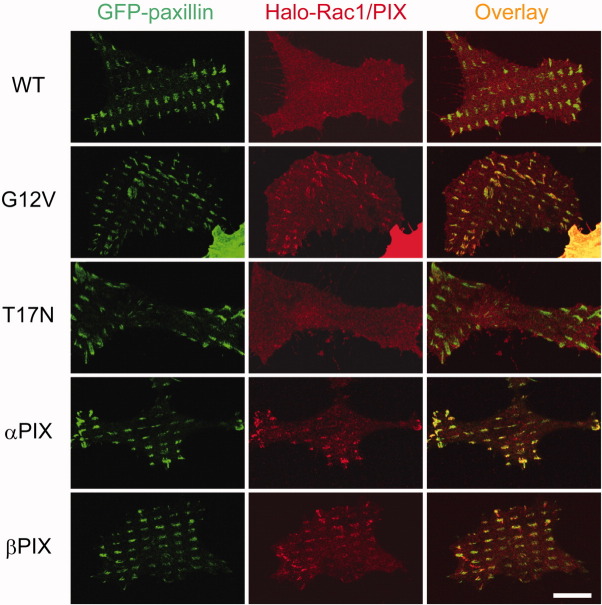
Activated Rac1 is accumulated in the FA zone mGFP-paxillin and Rac1 or PIX (Halo-labeled) expressed in HeLa cells were imaged using the photon-counting mode of a scanning laser confocal microscope. G12V as well as αPIX and βPIX exhibited clear accumulation in the FA zone marked by mGFP-paxillin. Accumulation of WT was much less than G12V and that of T17N was even less, suggesting that activated Rac1 tends to be accumulated more in the FA zone, consistent with the single-molecule observations, in which each individual G12V molecule exhibited much higher levels of temporary and long-term immobilization. Scale bar: 20 μm.

Accumulation of Rac1 and PIX molecules in the FA zone was inspected using the photon-counting mode of a scanning laser confocal microscope in the cells transfected with Halo-conjugaed molecules (Fig. 7). The expression levels were kept low (to ciucumvent possible overexpression artifacts) at levels where Rac1 and PIX signals were totally hidden in the noise by normal confocal microscopy without using the photon-counting mode, and similar to each other within ±50% (see Materials and Methods). Under these expression conditions, the accumulation of G12V in the FA zone, marked by mGFP-paxillin, was clearly visible (consistent with previous results by ten Klooster et al. [2006] and Wang et al. [2012], although they were obtained under overexpression conditions), whereas the accumulation of WT was much less, and that of T17N is hardly detected. Meanwhile, αPIX and βPIX are clearly concentrated in the FA zone. Consistent with these observatons, accumulation of WT in the FA zone has rarely been reported without its overexpression, except for a few reports [for example, see Chang et al.,^2007^]. These results suggest that WT-Rac1 molecules that entered the FA zone might become immobilized and accumulated in the FA zone after they are activated by GTP exchange factors such as PIX in the FA zone. As described, PIX molecules are immobilized and accumulated in the FA zone.

Since the expression of Rac1 and its mutants might affect the FA zone itself, we examined the sizes of the FA zone using such images shown in Fig. 7. We did not detect any signs of the changes of FA sizes (the longest diameter was measured; mean ± SD; WT, 3.1 ± 1.2; G12V, 3.2 ± 1.1; T17N, 3.5 ± 1.8; Tail, 3.3 ± 1.1 [μm]). In the case of single-molecule experiments, the expression levels of Rac1 and its mutants were less than those used here at least by a factor of 20 (doxycyclin was not added and leaky expression was used), and only 50–150 fluorescent Rac1 or mutant molecules were found on the entire basal PM at any moment (right after turning on the excitation laser before photobleaching took place). Therefore, it is concluded that expression of Rac1 and its mutants at this level will not change the FA structures and properties.

## Discussion

**Fig. 8:**
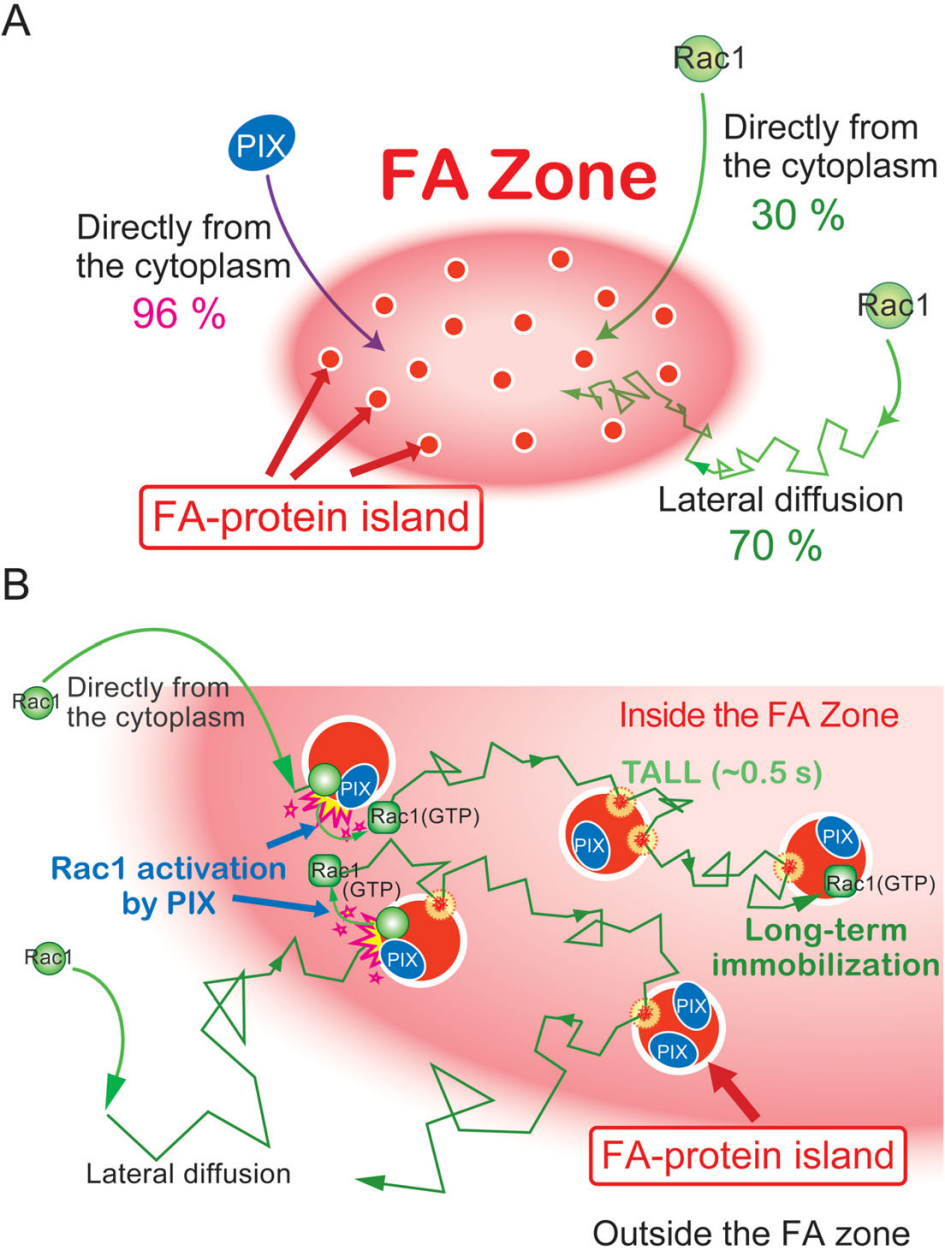
Schematic drawings for Rac1 and PIX recruitment processes to the FA-protein islands in the FA zone and their possible interactions **(Panel A)** The conclusions of the present research are summarized. Majorities of PIX molecules are directly recruited to the FA zone from the cytoplasm and immobilized there, whereas ≍70% of Rac1 (WT) molecules first arrive on the general inner surface of the PM and then recruited to the FA zone by translational diffusion on the cytoplasmic surface. **(Panel B)** Proposal for Rac1 recruitment to the FA-protein islands and their interactions with PIX. We propose that Rac1 molecules that entered the FA zone will be activated by the collision with PIX molecules as well as other GTP exchange factors bound on the FA-protein islands, and then will become more susceptible to immobilization on the islands, where they might activate p21-activated kinase (PAK, Rac1 effector) or might be deactivated by ArfGAP24 (Rac1 GAP).

Virtually, all PIX molecules were recruited to the FA zone directly from the cytoplasm. Since practically all PIX molecules became immobilized there without showing any sign of diffusion, they are likely to bind to the FA-protein islands directly and become immobilized there and work there (Fig. 8A).

In contrast, approximately 70% of Rac1 molecules that reached the FA zone first arrived on the general area of the inner PM surface, underwent diffusion there, and entered the FA zone (Fig. 8A). Rac1 molecules continue undergoing diffusion within the FA zone, and some of them might soon exit from the FA zone (Figs. 8A and 8B; Rac1 molecules that exited the FA zone might come back to the FA zone). Such pathways of Rac1 recruitment to the FA zone are possible because Rac1 molecules can undergo translational diffusion within the FA zone.

Approximately 30% of Rac1 molecules that entered the FA zone was directly recruited from the cytoplasm. Some of the directly recruited molecules were immobile in the FA zone right after they landed, but some were mobile, suggesting that Rac1 that directly arrived from the cytoplasm bound to both the FA-protein islands and the fluid-state membrane in the inter-island channels in the FA zone.

G12V exhibited much more frequent and longer TALLs and far more frequent long-term immobilization compared with the WT or T17N (Table I). These results suggest that some Rac1 molecules within the FA zone, in the course of their diffusion, might become activated by PIX immobilized on the FA-protein islands (Fig. 8B; of course other Rac1 GEFs including Tiam1 and DOCK180 might be involved in the Rac1 activation at the FA-protein islands [Huveneers and Danen,^2009^; Parsons et al.,^2010^; Wang et al.,^2012^], although the mechanism by which Tiam1 and Vav2 GEFs activate Rac1 might be more complicated than that by βPIX [ten Klooster et al.,^2006^; Wang et al.,^2012^]), and the activated Rac1 became more prone to the binding to the FA-protein islands, exhibiting temporary or long-term immobilization. Based on these observations, we propose a possibility in which, by diffusion in the outer peripheral region of the FA zone and also within the FA zone, the chances of Rac1′s being activated by its GEF(s) as well as its activating greater numbers of downstream molecules (typically PAK), which are probably immobilized in the FA-protein islands, will be enhanced, and this might be one of the most important biological reasons why majorities of Rac1 molecules are recruited to the FA zone via lateral diffusion on the PM.

The diffusion coefficients of WT and mutant Rac1 within the FA zone is smaller than those outside the FA zone, but only by a factor of two to four. This is consistent with the “Archipelago Model” previously proposed by us, in which the FA might still basically be a fluid membrane region where islands composed of various FA proteins are dotted, but where sufficient distances are maintained between the islands to allow the diffusion-based exchange of membrane molecules between the bulk membrane and the FA domain. The FA structure that allows fast diffusion of any membrane molecules, including FA-associated integrin β3 as well as non-FA phospholipids, transmembrane proteins, and GPI-anchored proteins, is a key feature of the archipelago model, because, this way, FA can be formed and disintegrated rapidly. Membrane-associated FA proteins including integrins and Rac1 could be rapidly assembled into FA-protein islands because they could enter and diffuse in the FA zone, and form many FA-protein islands simultaneously or become associated with preexisting islands simultaneously. FA-protein islands can be rapidly disassembled because FA-associated membrane molecules could be dissociated from each FA-protein island simultaneously, dispersed by lateral diffusion, and possibly internalized via clathrin-coated pits [Kawakami et al.,^2001^].

The present Rac1 and PIX results are consistent with the archipelago model, and directly showed how Rac1 and PIX are recruited from the cytoplasm. Furthermore, it suggests how Rac1 might be activated, bound to, and accumulated in the FA-protein islands. Such regulation mechanisms involving Rac1 will be important for dynamic formation and disintegration of FA-protein islands [ten Klooster et al.,^2006^; Wang et al.,^2012^]. The next key step in this line of research would be to directly observe Rac1 activation at the FA-protein islands at the level of single molecules, to directly image the FA-protein islands as well as the processes of their formation and disintegration by visualizing single molecules of FA proteins, and to understand how the islands are loosely assembled to form the FA zone.

## Materials and Methods

### Plasmid Construction

The cDNA encoding mGFP-paxillin was subcloned into the EBV-based episomal vector, pOsTet15T3, which carries the tetracycline-regulated expression units, the transactivator (rtTA2-M2) and the TetO sequence (a Tet-on vector). Halo7-Rac1 cDNA was generated by replacing the GFP sequence in the GFP-Rac1 cDNA (a generous gift from Dr. Klaus M. Hahn of the University of North Carolina) [Wu et al.,^2009^] with the Halo7 protein cDNA (Promega) and then subcloned into the pOsTet15T3 vector. The linker sequence encoding a 25-amino acid peptide, SGLRSRAQASSGGGGSGGGGQASNS, was then inserted between the Halo7 and Rac1 sequences. A point mutation was introduced to create the G12V and T17N constructs.

The Halo-αPIX cDNA was generated by replacing the Rac1 sequence in the Halo7-Rac1 sequence in the pOsTet15T3 vector with the αPIX sequence obtained from the GFP-αPIX cDNA (a generous gift from Dr. Arthur Weiss, the University of California San Francisco [Phee et al.,^2005^]). The cDNA encoding βPIX-Halo7 was generated by replacing the GFP sequence in the βPIX-GFP in the pEGFP-N1 vector (a generous gift from Dr. Arthur Weiss [Phee et al.,^2005^]) with the Halo-tag7 sequence and then by inserting a 45 base pairs linker (15 amino acids, with the sequence SGGGG x3). This sequence was then introduced into the pOsTet15T3 vector.

The cDNA encoding the Halo7-Tail was generated by replacing the GFP sequence in pEGFP-C1 vector with the Halo7 sequence, and then inserting the cDNA encoding the 11-amino acid C-terminus sequence of Rac1, VKKRKRKCLLL, at the C-terminus of the Halo7. All of the newly generated constructs were confirmed by DNA sequencing.

### Cell Culture, Transfection, and Fluorescence Labeling

HeLa cells were grown in Ham's F12 medium (Sigma-Aldrich), supplemented with 10% fetal bovine serum (Sigma-Aldrich), 100 units·ml^−1^ penicillin and 0.1 mg·ml^−1^ streptomycin (Gibco), and were transfected with various cDNAs by using LipofectAMINE LTX (Invitrogen), according to the manufacturer's recommendations. Cells were plated on 27-mm-ø coverslips (glass-base dishes from Iwaki, Funahashi, Japan), on which fibronectin was printed. Miro contact printing of fibronectin was performed as previously described [Singhvi et al.,^1994^; Katanosaka et al.,^2008^; Shibata et al.,^2012^]. To avoid perturbing the FA structure by overexpressing mGFP-paxillin and modifying the Rac1 signal, the doxycycline (which would induce higher expressions of these molecules) was not used (leaky expression was sufficient for single-molecule observations). To fluorescently label Halo-conjugated proteins, HeLa cells expressing one of these molecules (mGFP-paxillin was always coexpressed) were incubated with 0.1 nM TMR-conjugated Halo-linker (Promega) at 37°C overnight (0.5 nM when laser scanning confocal microscopy was performed). Single-molecule observations were always performed in HAM's F12 medium containing 10% FBS. For photon-counting scanning laser confocal microscopy, 20 ng·ml^−1^ doxycycline and 0.5 nM TMR-conjugated Halo ligand were added to the cells 16 h after transfection and incubated for another 16 h. After the cells were washed, they were fixed with 4% paraformaldehyde in PBS at room temperature for 1 h.

### TIRF Imaging of Single Molecules of Membrane-Associated Molecules and Assemblies of mGFP-paxillin

Fluorescently labeled molecules located on the bottom cell membrane (which faces the coverslip) were observed at 37°C in an atmosphere containing 5% CO_2_ in the stage chamber (Tokai Hit, Fujinomiya, Japan), using a home-built objective lens–type TIRF simultaneous, two-color microscope based on an inverted microscope (Nikon, Tokyo, Japan, ECLIPSE Ti-E), operated at 60 frames·s^−1^, as described previously [Murakoshi et al.,^2004^; Koyama-Honda et al.,^2005^; Shibata et al.,^2012^]. Although a simultaneous two-color microscope system was used, for ease of operation, multimolecular observations of mGFP-paxillin and single-molecule observations of membrane molecules (TMR) were performed alternatingly. This way, we did not need to make any subtle adjustments of the laser powers for each observation field, to avoid the leakage of the strong multimolecular fluorescent signal of mGFP-paxillin into the TMR channel. Briefly, mGFP-paxillin was first observed at 60 frames·s^−1^ for a few seconds, and subsequently, single membrane molecules (TMR) were observed at 60 frames·s^−1^ for a few to 16.7 s. The changes in the mGFP-paxillin images over several seconds were too small to detect under our experimental conditions. Single fluorescent-molecule images of membrane-associated molecules were then superimposed on the mGFP-paxillin images and analyzed as described previously [Koyama-Honda et al.,^2005^].

The methods for obtaining the trajectories of membrane-associated molecules, generating the plots of the mean-square displacement (MSD) versus time interval, and calculating the diffusion coefficient were described previously [Kusumi et al.,^1993^; Umemura et al.,^2008^; Shibata et al.,^2012^]. All of the single-molecule spots in the obtained images were detected, and those that were detectable for durations longer than 11 frames (183 ms) were quantitatively analyzed [Kusumi et al.,^1993^; Umemura et al.,^2008^], except for the analysis evaluating the durations of the residencies inside and outside the FA zone (see Results).

TALL events were detected in single-molecule tracking trajectories, by using the algorithm developed by Sahl et al. [2010], as described previously [Shibata et al.,^2012^]. False TALL detection occurred in the computer-generated simple-Brownian trajectories, which represented 2–4% of the total length of the trajectories. Using this program, all of the single-molecule trajectories obtained at 60 frames·s^−1^ were classified into the three modes of motion: (1) the all-time mobile mode, (2) the mobile + TALL mode, and (3) the all-time immobile mode.

### Defining the Boundary of the FA

Fluorescent images of mGFP-paxillin (8-bit grayscale data, 480 × 480 pixels, 50.0 × 50.0 nm^2^/pixel) were binarized, using adaptive (local) thresholding, in which each image was divided into 256 (16 × 16) blocks of 30 × 30 pixels, and then threshold values were determined for each pixel [Nakagawa and Rosenfeld,^1979^; Otsu,^1979^; Shibata et al.,^2012^]. The outer-most row of white color pixels in a binary image was considered as the outline of an FA zone, and was used to determine whether a single molecule is located inside or outside the FA zone.

### The Distributions of the Places where the Rac1 and PIX Molecules Arrive from the Cytoplasm on the Inner PM Surface with Respect to the FA Zone's Outer Contour

The distributions of the places where the Rac1 and PIX molecules arrive from the cytoplasm on the inner PM surface with respect to the FA zone's outer contour (Fig. 2) were determined in the following way. The observed area on the inner surface was sectioned into 200-nm-wide strips parallel to the FA boundaries (contours). The distance from the place (*x*-*y* coordinate) that Rac1 and PIX molecules landed on the cytoplasmic surface of the PM to the closest FA boundaries (contours) was measured and plotted in the strips formed around the closest FA boundaries. This was done for all molecules that arrived on the inner PM surface during observation. The area of each 200-nm strip was measured, and the number density of the molecules' arrival locations for each strip was determined by dividing the number of arrived molecules in the strip by the area size of the strip. For producing the histograms shown in Fig. 2, the number density was normalized to its average value outside the FA zone (between 1000 and 2000 nm from the FA boundaries).

### Photon-Counting Scanning Laser Confocal Microscopy

HeLa cells simultaneously expressing one of the Halo-conjugated molecules (WT, G12V, T17N, αPIX, and βPIX; TMR-labeled) and mGFP-paxillin were, after paraformaldehyde fixation, observed at room temperature, using the photon-counting mode of a Zeiss (Jena, Germany) LSM780 confocal microscope (63× 1.40 NA objective). For assessing the signal from the whole cell (needed to evaluate the number of labeled molecules in the entire cell), image slices were taken every 1 μm HeLa cells from the bottom to the top of the cell, and the total signal intensity was obtained using IMARIS software (Bitplane, Zürich, Switzerland). The background signal was obtained by using nontransfected cells after the addition of 0.5 nM TMR and the subsequent washing, followed by paraformaldehyde fixation.
