# Supplementary material for: Rac1 Recruitment to the Archipelago Structure of the Focal Adhesion through the Fluid Membrane as Revealed by Single-Molecule Analysis
Source: Cytoskeleton (Hoboken). 2013 Mar 5;70(3):161–77. doi: 10.1002/cm.21097 (PMC3627312; doi:10.1002/cm.21097)
Supplement: Supplementary file 1 [file cm0070-0161-SD1.pdf]

Supporting Information

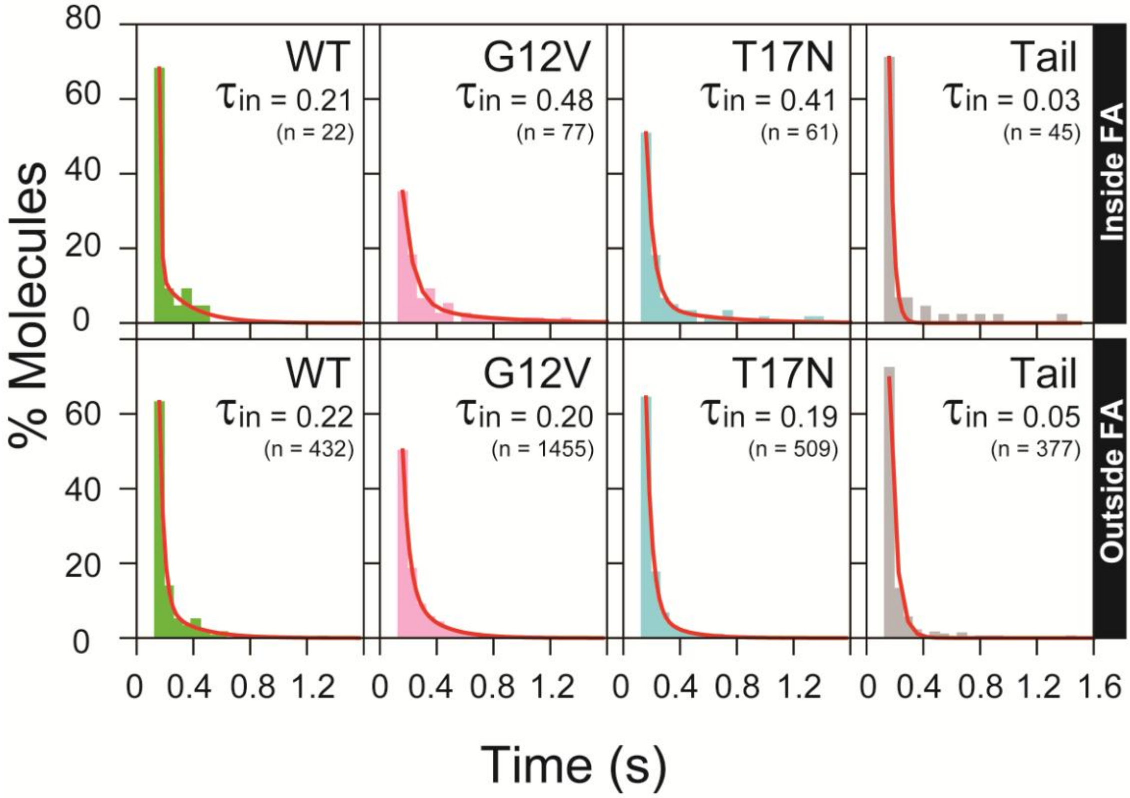

Fig. S1. The distribution of the TALL duration of Rac1 molecules in the FA zone, providing 0.21~0.48 after correction for photobleaching.

The distributions of Rac1 were fitted by double exponential functions, except for that of Tail, which was well fitted by a single exponential function. The shorter decay time constants for the full-length Rac1 molecules are similar to the decay time constants of Tail, suggesting that these shorter time constants are due to spurious noise in TALL analysis.

Supplementary Movies

Movie S1

A single Rac1 (WT) molecule diffusing in the basal PM, observed at a frame rate of 60 Hz (observation period = 9.2 s; slowed from real time by 2-fold). Clear green spots represent single WT molecules, and blue areas represent the FA zones labeled by mGFP-paxillin. The trajectory of one of the Rac1 molecules is superimposed on its image. Scale bar=1 μm.

Movie S2

A single G12V molecule diffusing in the basal PM, observed at a frame rate of 60 Hz (observation period = 7.1 s; slowed from real time by 2-fold). See the caption to Movie S1 for details.

Movie S3

A single αPIX molecule suddenly appears on the basal PM and immobilized right after its appearance, probably representing the touch down from the cytoplasm to an FA-protein island. Observed at a frame rate of 60 Hz (observation period = 26.1 s; slowed from real time by 2-fold). See the caption to Movie S1 for further details.
